# *ASXL1* mutated chronic myelomonocytic leukemia in a patient with familial thrombocytopenia secondary to germline mutation in *ANKRD26*

**DOI:** 10.1038/bcj.2015.41

**Published:** 2015-05-22

**Authors:** J Perez Botero, J L Oliveira, D Chen, K K Reichard, D S Viswanatha, P L Nguyen, R K Pruthi, J Majerus, P Gada, N Gangat, A Tefferi, M M Patnaik

**Affiliations:** 1 Division of Hematology, Department of Medicine, Mayo Clinic, Rochester, MN, USA; 2 Department of Laboratory Medicine and Pathology, Mayo Clinic, Rochester, MN, USA; 3 Minnesota Hematology and Oncology, Minneapolis, MN, USA

Thrombocytopenia 2 (THC2), a non-syndromic, autosomal dominant thrombocytopenia, is caused by mutations in the *ANKRD26* gene, located on chromosome 10p12.^1^ Thus far, 12 heterozygous single nucleotide substitutions in the 5′-untranslated region of the *ANKRD26* gene have been described in 21 families.^2, 3^ Affected patients usually present with moderate thrombocytopenia with normal platelet size and normal mean platelet volumes. Many patients were reported to have an α-granule deficiency as detected by immunofluorescent labelling without consistent defects in platelet aggregation studies. Evidence for dysmegakaryopoiesis has been observed in a small number of patients who have undergone bone marrow biopsies. Surprisingly, a small subset of individuals can have elevated hemoglobin levels and leukocyte counts, without a clear explanation.^3^

Germline mutations in *RUNX1* (ref. 4) and recently *ETV6* (ref. 5) have been associated with familial thrombocytopenias with an inherent predisposition to myeloid neoplasms. This phenomenon has also been described in patients with germline *ANKRD26* mutations. In an extended series of 118 patients with Thrombocytopenia 2 (THC2), 10 developed myeloid neoplasms: acute myeloid leukemia-4 (age at onset, 40–60 years), myelodysplastic syndrome-4 (MDS) (age at onset, 35–70 years) and chronic myeloid leukemia-2 (age at onset, 30–65 years).^6^ Two additional patients: a pregnant woman with acute myeloid leukemia and her mother with myelodysplastic syndrome-4, have also been described.^7^

We describe a 62-year-old male who had been referred to our institution for evaluation of monocytosis and chronic worsening thrombocytopenia. He had been diagnosed with idiopathic thrombocytopenia purpura at age 16 and had remained largely asymptomatic. A bone marrow biopsy was performed at age 35, which upon review demonstrated small hypolobated and bilobed megakaryocytes without additional dysplasia (Figure 1a). Throughout his life his platelet counts ranged from 50 to 60 × 10(9)/l without additional blood count abnormalities. Recently, his 32-year-old daughter had also been diagnosed with idiopathic thrombocytopenia purpura. A routine complete blood count at age 65 demonstrated a hemoglobin of 16.5 g/dl, mean corpuscular volume 81.2 fL, white blood cell count 7.8 × 10(9)/l, absolute monocyte count 4.2 × 10(9)/l, absolute neutrophil count 1.5 × 10(9)/l and platelets 25 × 10(9)/l. A repeat bone marrow biopsy demonstrated persistent dysmegakaryopoiesis, with bone marrow and peripheral blood monocytosis and no increase in myeloblasts, thus fulfilling the 2008 WHO (World Health Organization) criteria for diagnosis of chronic myelomonocytic leukemia-1 (Figures 1b and d). Next-generation sequencing demonstrated a frameshift *ASXL1* c.2290delC mutation. Transmission platelet electron microscopy showed minimally reduced dense granules (Figures 2a and b) and the platelet glycoprotein flow cytometry was normal. Sanger sequencing of the *ANKRD26* gene demonstrated a well-described heterozygous THC2 mutation; c.-116 C>T in the 5'UTR (Figure 2c).^3^

As illustrated in this case, an unknown proportion of patients diagnosed with idiopathic thrombocytopenia purpura in fact have familial thrombocytopenias, often with an inherent predisposition for myeloid neoplasms. To the best of our knowledge, this is the first case of chronic myelomonocytic leukemia-1 reported in a patient with THC2.^3^ Recently, there was a case report of a patient with a somatic *CBL* mutation who developed chronic myelomonocytic leukemia-1 in the setting of a familial thrombocytopenia secondary to a germline *RUNX1* mutation.^8^ Several patients with THC2 have been noted to have unexplained erythrocytosis and leukocytosis, and additional investigation to further the understanding of this association is needed. Somatic *ASXL1* mutations resulting in predicted frameshift or truncation coding alterations are frequent in chronic myelomonocytic leukemia-1 (~40%) and are prognostically detrimental, independently predicting for an inferior overall survival.^9, 10^ Affected patients tend to display more severe anemia and show a higher incidence of leukocytosis and monocytosis.^11^ We are currently evaluating our patient for an allogeneic stem cell transplant.

## Figures and Tables

**Figure 1:**
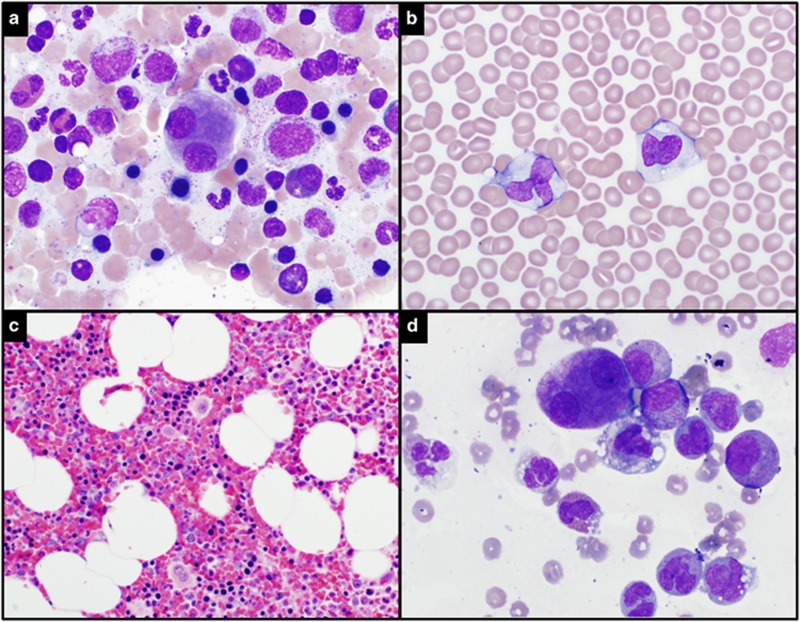
Peripheral blood and bone marrow aspirate and biopsy. (**a** and **d**) Bone marrow aspirate smears (**a**, 1987; **d**, 2015) reveal cytologically dysplastic megakaryocytes. (**b**) Peripheral blood smear in 2015 demonstrates normal appearing platelets and monocytes ( × 400). (**c**) Bone marrow core biopsy in 2015 shows cellular bone marrow with increased small megakaryocytes ( × 200).

**Figure 2:**
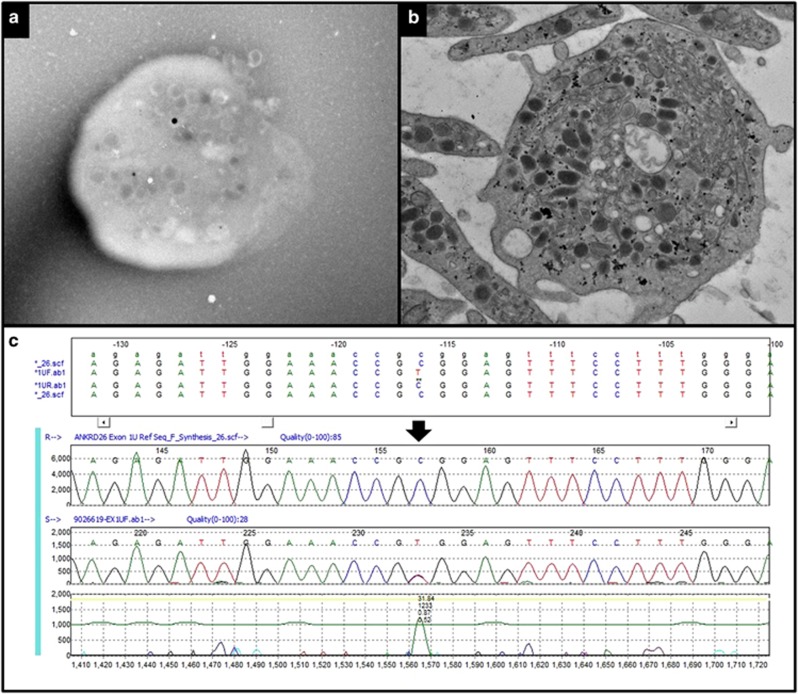
(**a**) Platelet whole mount electron microscopy shows slightly decreased dense granules. (**b**) Platelet thin section electron microscopy demonstrates occasional large platelets with complex canalicular system (on the right side) and normal alpha granules. (**c**) Sanger sequencing alignment with c.-116 C>T (arrow) in the 5′UTR of ANKRD26 gene.
